# Association of Platelet–Albumin–Bilirubin and Albumin–Bilirubin Scores with Angiographic No-Reflow in Patients with ST-Segment Elevation Myocardial Infarction Undergoing Primary PCI

**DOI:** 10.3390/jcm15207871

**Published:** 2026-10-12

**Authors:** Bülent Özlek, Veysel Ozan Tanık, Alperen Taş, Sena Tokatlı, Mehmet Taha Özkan, Kürşat Akbuğa, Yusuf Bozkurt Şahin, Murat Akdoğan, Çağatay Tunca, Betül Akıncıoğlu

**Affiliations:** 1 Department of Cardiology, School of Medicine, Muğla Sıtkı Koçman University, 48000 Muğla, Turkey; 2 Department of Cardiology, Lokman Hekim Akay Hospital, 06700 Ankara, Turkey; 3 Department of Cardiology, Kırşehir Training and Research Hospital, 40100 Kırşehir, Turkey; 4 Department of Cardiology, Ankara Etlik City Hospital, 06170 Ankara, Turkeymehmettahaozkan@gmail.com (M.T.Ö.);

**Keywords:** albumin–bilirubin score, no-reflow, platelet–albumin–bilirubin score, primary percutaneous coronary intervention, ST-segment elevation myocardial infarction

## Abstract

**Background**: The platelet–albumin–bilirubin (PALBI) and albumin–bilirubin (ALBI) scores integrate routinely available laboratory parameters with potential relevance to microvascular injury. We investigated their associations with angiographic no-reflow and incremental predictive value in patients with ST-segment elevation myocardial infarction (STEMI) undergoing primary percutaneous coronary intervention (PCI). **Methods**: This retrospective, single-center study included 1539 consecutive patients with STEMI undergoing primary PCI. No-reflow was defined by final TIMI flow < 3 and/or myocardial blush grade 0–1 in the absence of a mechanical cause. Associations of PALBI and ALBI with no-reflow were assessed using multivariable logistic regression. Discrimination, calibration, reclassification, internal bootstrap validation, and restricted cubic spline analyses were performed. **Results**: No-reflow occurred in 146 patients (9.5%). Both PALBI (adjusted odds ratio [aOR] per 0.1-point increase, 1.315; 95% confidence interval [CI], 1.228–1.408; *p* < 0.001) and ALBI (aOR, 1.165; 95% CI, 1.115–1.218; *p* < 0.001) were independently associated with no-reflow. PALBI showed greater discrimination than ALBI (AUC, 0.722 vs. 0.681; DeLong *p* = 0.018). Adding PALBI to the clinical model increased the AUC from 0.688 to 0.785 (ΔAUC = 0.098; *p* = 0.001), with significant NRI (0.777) and IDI (0.102), and improved the Brier score from 0.083 to 0.073. The optimism-corrected AUC was 0.762. PALBI demonstrated a non-linear association with no-reflow (*p* for non-linearity <0.001), whereas ALBI showed a predominantly linear association. **Conclusions**: Higher PALBI and ALBI scores were independently associated with angiographic no-reflow after primary PCI in STEMI. PALBI demonstrated stronger discrimination and incremental predictive performance; however, external validation and prospective evaluation are required to determine its potential clinical utility.

## 1. Introduction

Current international guidelines recommend primary percutaneous coronary intervention (PCI) as the preferred reperfusion strategy for patients with ST-segment elevation myocardial infarction (STEMI) when it can be performed within the recommended time frame [1,2]. Despite guideline-directed timely reperfusion and successful restoration of infarct-related artery patency, adequate myocardial microvascular reperfusion is not invariably achieved. The no-reflow phenomenon therefore remains an important complication of primary PCI and has been associated with larger infarct size, impaired recovery of left ventricular function, adverse ventricular remodeling, heart failure, and increased mortality [3,4,5]. Identification of patients at increased risk of no-reflow may provide additional information for risk characterization; however, whether such predictive information can be obtained within a clinically relevant time frame and translated into effective preventive strategies or improved clinical outcomes remains uncertain.

No-reflow is a complex process reflecting coronary microvascular injury, and numerous demographic, clinical, laboratory, and angiographic variables have been investigated as potential predictors [6,7,8]. However, the predictive ability of individual parameters is variable, and currently available risk models differ considerably in their composition and performance. Consequently, there remains interest in simple, objective, and readily available biomarkers that may provide additional information beyond conventional clinical risk factors.

The albumin–bilirubin (ALBI) score was originally developed as an objective index of hepatic functional reserve and is calculated from two routinely available laboratory parameters, serum albumin and bilirubin [9]. More recently, ALBI has attracted interest beyond liver disease, with accumulating evidence linking the score to prognosis in several cardiovascular conditions [10,11]. The platelet–albumin–bilirubin (PALBI) score incorporates platelet count in addition to albumin and bilirubin and may therefore provide complementary information derived from three routinely measured laboratory parameters [12]. Both scores are derived from routinely measured laboratory parameters and do not require additional biomarker testing; however, their potential use for preprocedural risk assessment would depend on timely availability of all component laboratory results.

Despite increasing interest in ALBI- and PALBI-based indices in cardiovascular risk stratification, their role in coronary no-reflow remains insufficiently characterized. To our knowledge, the association between PALBI and no-reflow has not been specifically investigated in patients with STEMI undergoing primary PCI, and whether PALBI provides incremental predictive information beyond established clinical variables remains unknown. Moreover, the comparative predictive performance of PALBI and ALBI for angiographic no-reflow in this setting has not been established. Accordingly, the present study aimed to investigate the association between PALBI and angiographic no-reflow in patients with STEMI undergoing primary PCI and to assess its discriminative and incremental predictive value beyond conventional clinical variables. In parallel, we evaluated the association and predictive performance of ALBI and compared the predictive characteristics of the two scores.

## 2. Methods

### 2.1. Study Design and Population

This retrospective, single-center observational study included consecutive adult patients with STEMI who underwent primary PCI at a tertiary-care center between January 2022 and October 2025. Patients were identified through the institutional electronic medical records and the cardiac catheterization laboratory database. Clinical characteristics, cardiovascular risk factors, laboratory measurements, echocardiographic findings, procedural and angiographic data, and in-hospital outcomes were retrospectively obtained from the hospital information system and archived patient records. STEMI was defined according to the diagnostic criteria applicable during the study period, based on a clinical presentation consistent with acute myocardial ischemia accompanied by diagnostic ST-segment elevation on the 12-lead electrocardiogram and subsequent evidence of myocardial necrosis. Only patients treated with primary PCI without preceding fibrinolytic therapy were included in the analysis. Patients received guideline-directed standard medical therapy for STEMI in addition to primary PCI, according to contemporary recommendations and routine institutional practice during the study period. Patients were excluded in the presence of chronic liver disease, including cirrhosis or active hepatitis; active infection or sepsis at the time of admission; systemic inflammatory or autoimmune disease; severe hematological disorders substantially affecting platelet count or hemoglobin levels; known active malignancy or ongoing cancer treatment; or missing laboratory or angiographic data required for calculation of the study variables or ascertainment of the primary outcome.

### 2.2. Data Collection and Clinical Definitions

Recorded clinical variables included age, sex, hypertension, diabetes mellitus (DM), dyslipidemia, smoking status, family history of coronary artery disease, previous myocardial infarction, previous PCI, previous coronary artery bypass grafting, chronic kidney disease (CKD), cardiac arrest at admission, and pre-admission medications documented in the medical records. Pre-admission medications were defined as medications that patients were documented to be using before the index STEMI presentation and did not represent medications administered as part of acute STEMI treatment after hospital arrival. Hypertension, DM, and dyslipidemia were identified from documented medical history, previous physician diagnosis, or ongoing disease-specific treatment recorded in the medical files. CKD was defined by a pre-existing documented diagnosis of CKD in the electronic medical records together with an admission estimated glomerular filtration rate (eGFR) < 60 mL/min/1.73 m^2^, consistent with the GFR threshold used in contemporary KDIGO classification [13]. CKD stage was not systematically documented in the retrospective records and therefore could not be reliably ascertained. Smoking status was based on the smoking history documented at admission. Cardiac arrest at presentation was defined according to the clinical documentation available in the emergency and hospitalization records. Symptom-to-balloon time was calculated as the interval between the documented onset of ischemic symptoms and balloon inflation or device-mediated restoration of flow during primary PCI. Total length of hospital stay was calculated from hospital admission to discharge or in-hospital death. In-hospital mortality was defined as all-cause death occurring during the index hospitalization.

### 2.3. Laboratory and Echocardiographic Assessment

With the exception of peak troponin, all laboratory parameters included in the study were measured from blood samples obtained at emergency department admission before primary PCI as part of routine clinical care. No post-PCI measurements of platelet count, serum albumin, or total bilirubin were used for calculation of the PALBI or ALBI scores or included in the principal analyses of these scores. However, although the samples were obtained before PCI, the retrospective records did not permit reliable determination of whether all laboratory results required for calculation of PALBI and ALBI were available to the treating interventional cardiologist before initiation of PCI. The variables evaluated included white blood cell (WBC) count, hemoglobin, platelet count, serum creatinine, eGFR, blood glucose, serum albumin, total bilirubin, lipid parameters, and other laboratory variables included in the study dataset. Peak troponin was defined separately as the highest troponin concentration recorded among serial measurements obtained during the index hospitalization as part of routine clinical care. Renal function was represented principally by eGFR in the adjusted analyses because of its close relationship with serum creatinine and CKD. Left ventricular ejection fraction (LVEF) was derived from transthoracic echocardiographic assessment performed at initial presentation, either in the emergency department or in the cardiac catheterization laboratory before primary PCI, using the value documented in the corresponding echocardiography report.

### 2.4. Calculation of ALBI and PALBI Scores

The ALBI and PALBI scores were calculated retrospectively from admission laboratory measurements obtained from blood samples collected before primary PCI, using their established equations. ALBI incorporates serum albumin and total bilirubin, whereas PALBI additionally incorporates platelet count.

The ALBI score was calculated according to the following formula [9]:

ALBI score = [0.66 × log_10_ bilirubin (μmol/L)] − [0.085 × albumin (g/L)].

The PALBI score was calculated according to the following formula [12]:

PALBI score = 2.02 × log_10_ bilirubin (μmol/L) − 0.37 × [log_10_ bilirubin (μmol/L)]^2^ − 0.04 × albumin (g/L) − 3.48 × log_10_ platelet count (×10^3^/μL) + 1.01 × [log_10_ platelet count (×10^3^/μL)]^2^.

When bilirubin and albumin measurements were recorded in conventional units, values were converted to μmol/L and g/L, respectively, before score calculation. Both ALBI and PALBI were analyzed as continuous variables, with higher numerical (i.e., less negative) values representing higher scores.

### 2.5. Coronary Angiography and Primary PCI

Primary PCI was performed according to standard interventional practice and at the discretion of the treating interventional cardiologist. Tirofiban administration was performed at the discretion of the treating interventional cardiologist and was not restricted to patients who subsequently met the study definition of no-reflow. In the retrospective dataset, the specific indication and timing of tirofiban administration were not systematically recorded; therefore, we could not reliably distinguish administration for treatment of no-reflow from administration for other intraprocedural thrombotic indications. Angiographic and procedural characteristics were obtained retrospectively from catheterization laboratory records and archived angiographic reports. The culprit coronary artery was categorized as the left anterior descending artery (LAD), left circumflex artery (LCx), right coronary artery (RCA), obtuse marginal (OM) branch, diagonal branch, or ramus intermedius, as recorded in the procedural documentation. Procedural variables included culprit vessel, stent diameter, stent length, and other angiographic characteristics available in the study database.

### 2.6. Definition of the No-Reflow Phenomenon

No-reflow was defined as inadequate epicardial and/or myocardial tissue reperfusion after restoration of patency of the infarct-related artery in the absence of an identifiable mechanical cause of impaired coronary flow [3]. Angiographic reperfusion was assessed by the treating interventional cardiologists during the index procedure using the Thrombolysis in Myocardial Infarction (TIMI) flow grade and myocardial blush grade (MBG). No-reflow was considered present in patients with a final TIMI flow grade < 3 and/or severely impaired myocardial blush (MBG 0–1), despite successful treatment of the culprit epicardial lesion [3,14]. Before classification as no-reflow, identifiable mechanical causes of impaired coronary flow, including residual flow-limiting stenosis, coronary dissection, epicardial vasospasm, or distal epicardial vessel obstruction, were excluded [3,14]. Patients with preserved epicardial and myocardial reperfusion were classified as having normal reflow. Given the retrospective design of the study, no-reflow status was determined from the angiographic assessment documented by the treating interventional cardiologists in the catheterization laboratory records and procedural reports; no prospective core-laboratory adjudication was performed. Individual final TIMI flow and MBG grades were not retained as separate analyzable variables in the study dataset.

### 2.7. Study Outcomes

The primary outcome was angiographic no-reflow following primary PCI. The primary objective was to determine whether the PALBI score was independently associated with the occurrence of no-reflow and to evaluate its discriminative and incremental predictive performance beyond conventional clinical variables. The association and predictive performance of the ALBI score were evaluated in parallel as a secondary study objective. Additional in-hospital outcomes included all-cause in-hospital mortality and length of hospital stay. These outcomes were compared according to no-reflow status as descriptive secondary outcomes and were not incorporated into the definition of the primary endpoint.

### 2.8. Ethical Considerations

The study protocol was reviewed and approved by the Ankara Etlik City Hospital No. 2 Scientific Research Evaluation and Ethics Committee (approval date and number: 4 November 2025/AEŞH-BADEK2-2025-563). The study was conducted in accordance with the principles of the Declaration of Helsinki and applicable institutional and national regulations. Owing to the retrospective observational design and the use of routinely collected clinical data, the requirement for written informed consent was waived by the ethics committee. Patient data were analyzed in a de-identified/confidential manner in accordance with institutional data-protection requirements.

### 2.9. Statistical Analysis

All statistical analyses were performed using IBM SPSS Statistics for Windows, version 26.0 (IBM Corp., Armonk, NY, USA) and Python (version 3.10). The distribution of continuous variables was assessed before comparative analyses. Because the continuous variables were predominantly non-normally distributed, they are presented as the median and interquartile range (IQR) and were compared between patients with and without no-reflow using the Mann–Whitney U test. Categorical variables are expressed as numbers and percentages and were compared using Pearson’s chi-square test or Fisher’s exact test, as appropriate. All statistical tests were two-sided, and a *p* value <0.05 was considered statistically significant. The association of PALBI and ALBI scores with the occurrence of no-reflow was initially evaluated using univariable binary logistic regression. To facilitate clinically interpretable effect estimates and avoid inflated odds ratios arising from the numerical scale of the scores, odds ratios (ORs) for PALBI and ALBI are expressed per 0.1-point increase. Other continuous covariates were rescaled to clinically meaningful increments where appropriate, including age per 10 years, symptom-to-balloon time per 30 min, LVEF per 5 percentage points, eGFR per 10 mL/min/1.73 m^2^, blood glucose per 10 mg/dL, and stent length per 5 mm. Variables associated with no-reflow at *p* < 0.10 in univariable analyses were considered candidates for multivariable modeling; age and sex were retained a priori as core demographic covariates irrespective of univariable significance. This more inclusive threshold was selected to reduce the likelihood of excluding potentially relevant covariates on the basis of univariable significance alone. Because PALBI and ALBI are mathematically related indices and share constituent variables, they were evaluated in separate multivariable logistic regression models. Serum albumin and total bilirubin were not simultaneously entered with the corresponding composite scores to avoid mathematical coupling and collinearity. Similarly, because serum creatinine, CKD, and eGFR represent closely related measures of renal function, eGFR was retained in the primary multivariable models, whereas serum creatinine and CKD were excluded to minimize multicollinearity. The primary adjusted models therefore included age, sex, symptom-to-balloon time, LVEF, eGFR, WBC count, stent length, blood glucose, and culprit vessel, together with either PALBI or ALBI. Adjusted ORs (aORs) with 95% confidence intervals (CIs) are reported. This modeling strategy is consistent with the variables and exclusions reported in the final multivariable analysis. To examine the robustness of the associations and reduce dependence on any single covariate specification, a series of prespecified sensitivity models with progressively different clinical, laboratory, and procedural adjustment sets was constructed separately for PALBI and ALBI. Age and sex were included as core covariates, with additional models incorporating established or clinically plausible determinants of no-reflow, including DM, smoking, cardiac arrest on admission, blood glucose, WBC count, triglyceride level, CKD, symptom-to-balloon time, LVEF, stent length, and culprit vessel. PALBI and ALBI were again evaluated in separate models. Multicollinearity was assessed using the variance inflation factor (VIF); no relevant multicollinearity was observed across the sensitivity models. The discriminative ability of PALBI and ALBI for identifying patients with no-reflow was evaluated using receiver operating characteristic (ROC) curve analysis. Areas under the ROC curves (AUCs) were calculated with 95% CIs, and the AUCs of PALBI and ALBI were compared using DeLong’s test for correlated ROC curves. The optimal cutoff values for PALBI and ALBI were determined using the Youden index, defined as the point maximizing the sum of sensitivity and specificity. For assessment of incremental predictive value, a clinical reference model was constructed, including age, sex, symptom-to-balloon time, LVEF, eGFR, WBC count, stent length, blood glucose, and culprit vessel, with LAD used as the reference category. This model corresponded to the principal clinical and procedural covariate set used in the multivariable analyses but did not include PALBI or ALBI. The discrimination of the reference model was subsequently compared with that of models obtained after the separate addition of PALBI or ALBI, and changes in AUC (ΔAUC) were quantified and statistically tested. Model performance was further characterized beyond discrimination. Calibration was evaluated graphically by comparing predicted probabilities with observed no-reflow rates, and overall prediction error was quantified using the Brier score, with lower values indicating better predictive accuracy. The incremental predictive contribution of PALBI and ALBI beyond the clinical reference model was additionally explored using net reclassification improvement (NRI) and integrated discrimination improvement (IDI). CIs and statistical significance for NRI and IDI estimates were derived from 3000 bootstrap resamples. Internal validation was performed using bootstrap resampling to quantify model optimism and assess the stability of predictive performance. A total of 1000 bootstrap samples were generated with replacement from the original cohort; the prediction models were refitted within each bootstrap sample, and model performance was evaluated to obtain optimism-corrected estimates. Bootstrap-corrected discrimination and calibration measures were used to assess the extent to which apparent model performance might reflect overfitting in the derivation cohort. Finally, potential non-linear relationships between PALBI and ALBI and the odds of no-reflow were investigated using restricted cubic spline (RCS) logistic regression. Splines were modeled using four knots located at the 5th, 35th, 65th, and 95th percentiles of the respective score distributions. Adjusted spline models included age, sex, symptom-to-balloon time, LVEF, eGFR, and WBC count. The median value of each score was used as the reference (OR = 1). Overall associations and departures from linearity were evaluated separately using likelihood-ratio tests. Adjusted ORs and corresponding 95% CIs were plotted across the observed score range.

## 3. Results

### 3.1. Study Population and Baseline Characteristics

**Figure 1:**
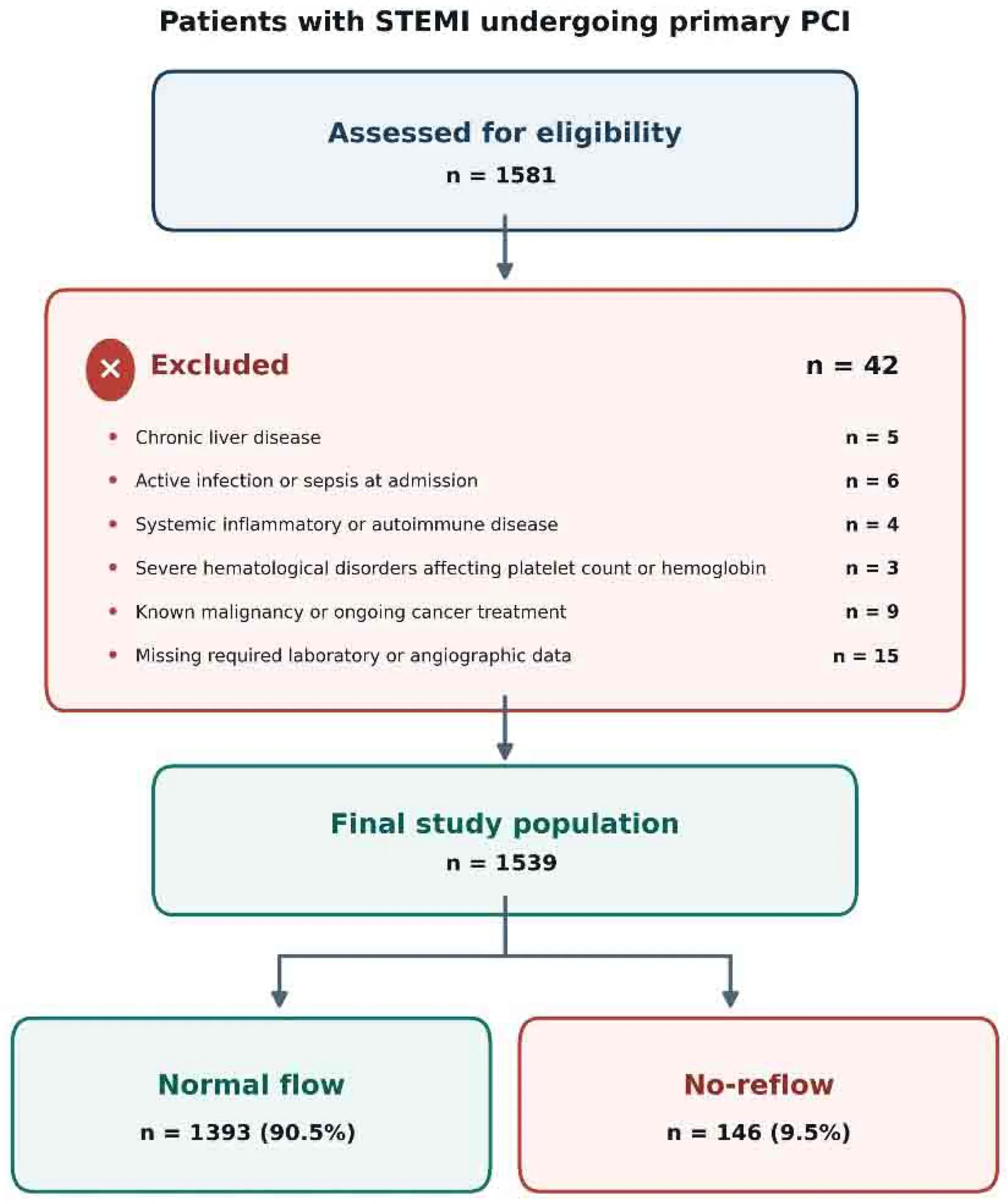
Flow diagram of patient selection and classification according to the occurrence of no-reflow. STEMI, ST-segment elevation myocardial infarction; PCI, percutaneous coronary intervention.

A total of 1581 patients with STEMI undergoing primary PCI were assessed for eligibility. After exclusion of 42 patients according to the prespecified exclusion criteria, 1539 patients constituted the final study population (Figure 1). No-reflow occurred in 146 patients, corresponding to an overall prevalence of 9.5%, whereas 1393 patients (90.5%) had preserved coronary flow. Overall, 1295 patients (84.1%) were male, and 120 patients (7.8%) died during hospitalization.

Baseline clinical, laboratory, angiographic, and in-hospital characteristics according to no-reflow status are presented in Table 1. Median age was 60.0 [52.0–67.8] years in patients with no-reflow and 58.0 [50.0–66.0] years in those without no-reflow (*p* = 0.064), while the proportion of male patients was similar between the groups (84.9 vs. 84.1%, *p* = 0.877). CKD was more frequent in the no-reflow group (5.5 vs. 1.7%, *p* = 0.006). Patients with no-reflow had lower LVEF (45.0% vs. 50.0%, *p* = 0.046), higher WBC counts (12.20 vs. 11.40 × 10^3^/mm^3^, *p* = 0.001), lower platelet counts (204.0 vs. 221.0 × 10^3^/mm^3^, *p* = 0.016), and lower eGFR (86.8 vs. 92.3 mL/min/1.73 m^2^, *p* < 0.001). Serum creatinine and total bilirubin concentrations were higher, whereas serum albumin concentrations were lower, among patients who developed no-reflow (all *p* < 0.001). Among patients who developed no-reflow, the culprit vessel was the RCA in 56 patients (38.4%), LAD in 51 patients (34.9%), LCx in 36 patients (24.7%), the OM branch in 2 patients (1.4%), and a diagonal branch in 1 (0.7%) patient.

Both composite scores differed significantly according to no-reflow status. The median PALBI score was −1.87 [−2.31 to −1.64] in the no-reflow group compared with −2.26 [−2.47 to −2.07] in the non-no-reflow group (*p* < 0.001), while the corresponding ALBI scores were −2.08 [−2.44 to −1.60] and −2.39 [−2.65 to −2.08], respectively (*p* < 0.001). Stent length was also greater in patients with no-reflow (20.0 [18.0–28.0] vs. 20.0 [16.0–24.0] mm, *p* = 0.004). In-hospital mortality was 13.7% in patients with no-reflow and 7.2% in those without no-reflow (*p* = 0.005). The median length of hospital stay was 3 [3,4] days in both groups (*p* = 0.122).

### 3.2. Logistic Regression Analyses

In univariable logistic regression analysis, both PALBI and ALBI were associated with no-reflow. For each 0.1-point increase in PALBI, the odds of no-reflow increased with an OR of 1.296 (95% CI 1.230–1.358; *p* < 0.001), while the corresponding OR for each 0.1-point increase in ALBI was 1.143 (95% CI 1.104–1.182; *p* < 0.001). Other variables meeting the prespecified *p* < 0.10 criterion included symptom-to-balloon time, age, LVEF, WBC count, eGFR, serum creatinine, blood glucose, serum albumin, total bilirubin, stent length, CKD, and culprit vessel (Table 2).

In the primary multivariable logistic regression models, PALBI and ALBI remained independently associated with no-reflow after adjustment for age, sex, symptom-to-balloon time, LVEF, eGFR, WBC count, stent length, blood glucose, and culprit vessel (Table 3). In the PALBI model, each 0.1-point increase in PALBI was associated with an aOR of 1.315 (95% CI 1.228–1.408; *p* < 0.001). In the corresponding ALBI model, each 0.1-point increase in ALBI was associated with an aOR of 1.165 (95% CI 1.115–1.218; *p* < 0.001). Symptom-to-balloon time was not statistically significant in the univariable analysis of the overall cohort (OR per 30 min, 1.030; 95% CI, 0.995–1.067; *p* = 0.095) but was independently associated with no-reflow in both multivariable models (aOR per 30 min, 1.063; 95% CI, 1.017–1.111; *p* = 0.007). Compared with LAD involvement, LCx culprit lesions were associated with higher odds of no-reflow in both the PALBI (aOR 1.936, 95% CI 1.060–3.535; *p* = 0.031) and ALBI (aOR 2.360, 95% CI 1.308–4.258; *p* = 0.004) models. Although sex was not associated with no-reflow in the unadjusted comparison, male sex was associated with higher odds of no-reflow in the fully adjusted PALBI model (aOR, 2.325; 95% CI, 1.117–4.842; *p* = 0.024). In an exploratory sequential adjustment analysis, the male-sex effect estimate increased progressively from OR 1.316 in the unadjusted model to 2.325 in the fully adjusted PALBI model, rather than changing abruptly after inclusion of a single covariate (Supplementary Table S1).

The associations of PALBI and ALBI with no-reflow remained consistent across the sensitivity analyses using different covariate adjustment sets (Table 4). Across Models A–E, the aORs per 0.1-point increase in PALBI ranged from 1.292 to 1.335, with all *p* values < 0.001. Corresponding aORs for ALBI ranged from 1.140 to 1.171, also with all *p* values < 0.001. Maximum variance inflation factors ranged from 1.108 to 1.289 across the models.

In an additional sensitivity analysis addressing the timing of predictor availability, a presentation-based reference model was constructed using age, sex, eGFR, WBC count, and admission blood glucose, while excluding angiographic and procedural variables and symptom-to-balloon time. The reference model yielded an AUC of 0.623 (95% CI, 0.572–0.675). Both ALBI (adjusted OR per 0.1-point increase, 1.129; 95% CI, 1.090–1.169; *p* < 0.001) and PALBI (adjusted OR per 0.1-point increase, 1.273; 95% CI, 1.206–1.343; *p* < 0.001) remained independently associated with no-reflow. Addition of ALBI increased the AUC to 0.711 (95% CI, 0.665–0.756; ΔAUC = 0.087; DeLong *p* < 0.001), whereas addition of PALBI increased the AUC to 0.745 (95% CI, 0.697–0.792; ΔAUC = 0.121; DeLong *p* < 0.001) (Supplementary Table S2).

### 3.3. Discrimination and Incremental Predictive Performance

**Figure 2:**
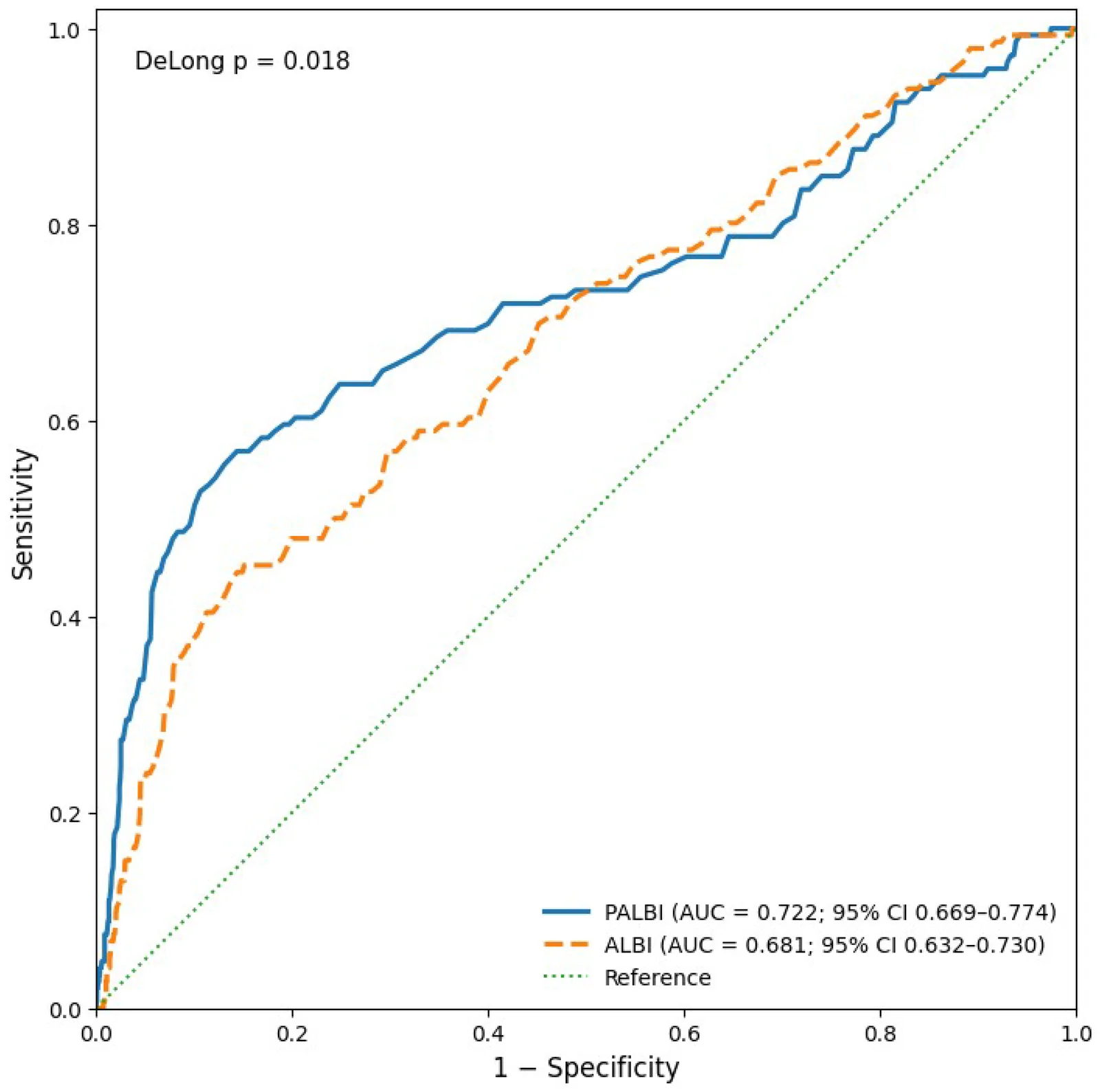
Receiver operating characteristic curves of PALBI and ALBI scores for predicting the no-reflow phenomenon. PALBI showed significantly greater discriminative ability than ALBI (AUC: 0.722 vs. 0.681; DeLong *p* = 0.018). AUC, area under the curve; CI, confidence interval; PALBI, platelet–albumin–bilirubin; ALBI, albumin–bilirubin.

**Figure 3:**
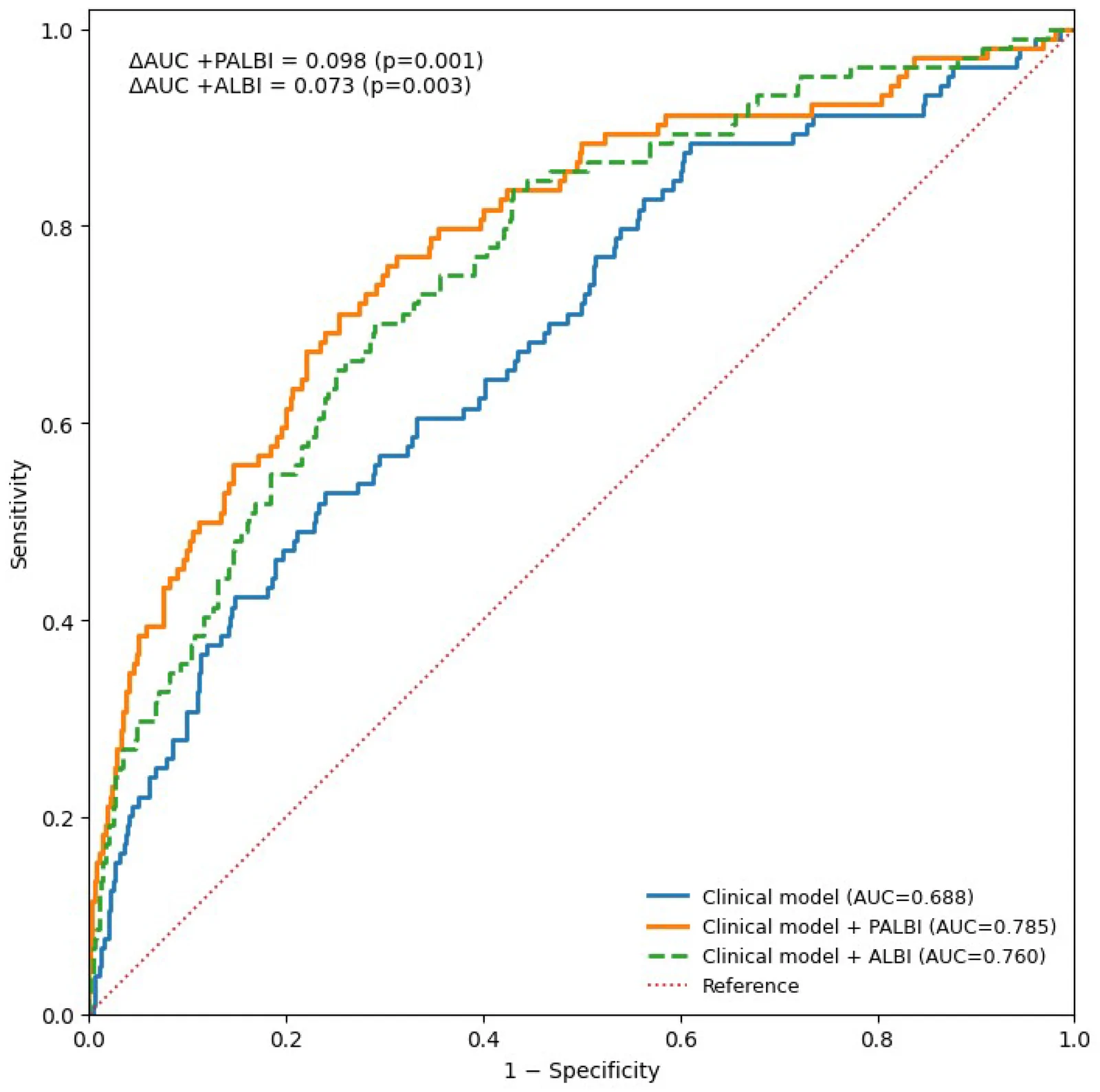
Incremental discriminative value of PALBI and ALBI over the clinical model for predicting no-reflow. Addition of PALBI and ALBI significantly improved the AUC of the clinical model (ΔAUC: 0.098, *p* = 0.001 and ΔAUC: 0.073, *p* = 0.003, respectively). AUC, area under the curve; PALBI, platelet–albumin–bilirubin; ALBI, albumin–bilirubin.

ROC analysis demonstrated an AUC of 0.722 (95% CI 0.669–0.774) for PALBI and 0.681 (95% CI 0.632–0.730) for ALBI in discriminating patients with and without no-reflow (Figure 2). The Youden-derived optimal cutoff for PALBI was −1.93, yielding a sensitivity of 56.8%, specificity of 85.6%, positive predictive value (PPV) of 29.3%, and negative predictive value (NPV) of 95.0%. The corresponding cutoff for ALBI was −1.88, with a sensitivity of 45.2%, specificity of 84.9%, PPV of 23.9%, and NPV of 93.7%. Direct comparison of the ROC curves using the DeLong test demonstrated a significant difference between the two AUCs (*p* = 0.018). The clinical reference model yielded an AUC of 0.688. Addition of PALBI increased the AUC to 0.785 (ΔAUC = 0.098, *p* = 0.001), whereas addition of ALBI increased the AUC to 0.760 (ΔAUC = 0.073, *p* = 0.003) (Figure 3). Reclassification analyses further demonstrated significant incremental predictive improvement with both scores. Addition of PALBI to the clinical model resulted in a category-free NRI of 0.777 (95% CI, 0.582–0.970; *p* < 0.001) and an IDI of 0.102 (95% CI, 0.072–0.134; *p* < 0.001). Addition of ALBI yielded an NRI of 0.565 (95% CI, 0.359–0.751; *p* < 0.001) and an IDI of 0.061 (95% CI, 0.038–0.085; *p* < 0.001). When PALBI was added to the clinical model already containing ALBI, the NRI was 0.489 (95% CI, 0.298–0.685; *p* < 0.001) and the IDI was 0.041 (95% CI, 0.024–0.059; *p* < 0.001).

### 3.4. Calibration and Internal Validation

**Figure 4:**
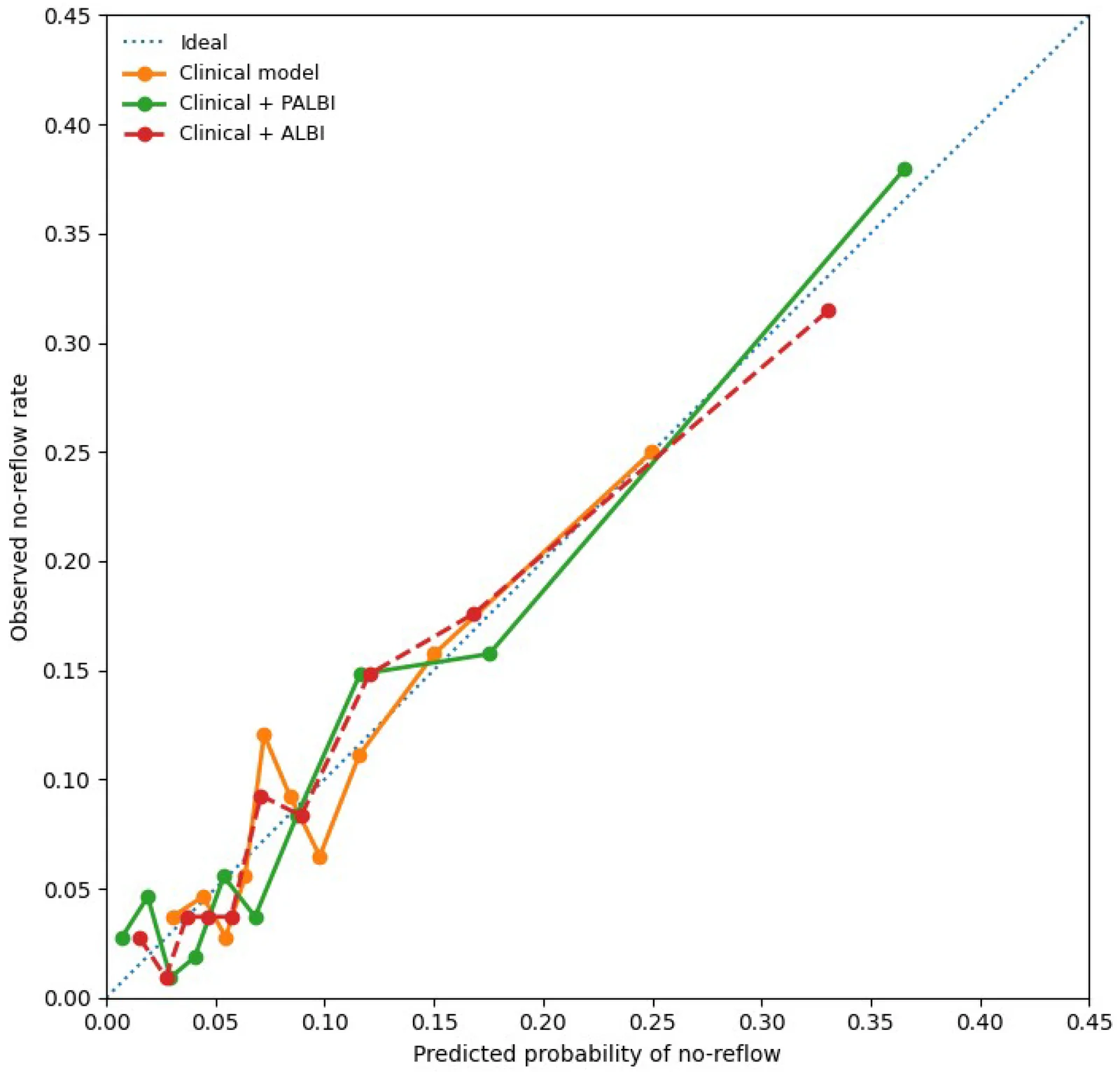
Calibration plots of the clinical model and models incorporating PALBI or ALBI for predicting no-reflow. The corresponding Brier scores were 0.083 for the clinical model, 0.073 for the clinical + PALBI model, and 0.077 for the clinical + ALBI model. Lower Brier scores indicate better overall predictive accuracy. PALBI, platelet–albumin–bilirubin; ALBI, albumin–bilirubin.

Calibration plots for the clinical reference model and the models incorporating PALBI or ALBI are presented in Figure 4. The Brier score was 0.083 for the clinical model, 0.073 for the clinical model plus PALBI, and 0.077 for the clinical model plus ALBI.

Bootstrap internal validation demonstrated persistence of model discrimination after correction for optimism. The optimism-corrected AUC was 0.651 for the clinical reference model, 0.762 for the clinical + PALBI model, and 0.733 for the clinical + ALBI model. The corresponding optimism-corrected Brier scores were 0.085, 0.076, and 0.080, respectively. Optimism-corrected calibration slopes were 0.703 for the clinical model, 0.848 for the clinical + PALBI model, and 0.821 for the clinical + ALBI model.

### 3.5. RCS Analyses

**Figure 5:**
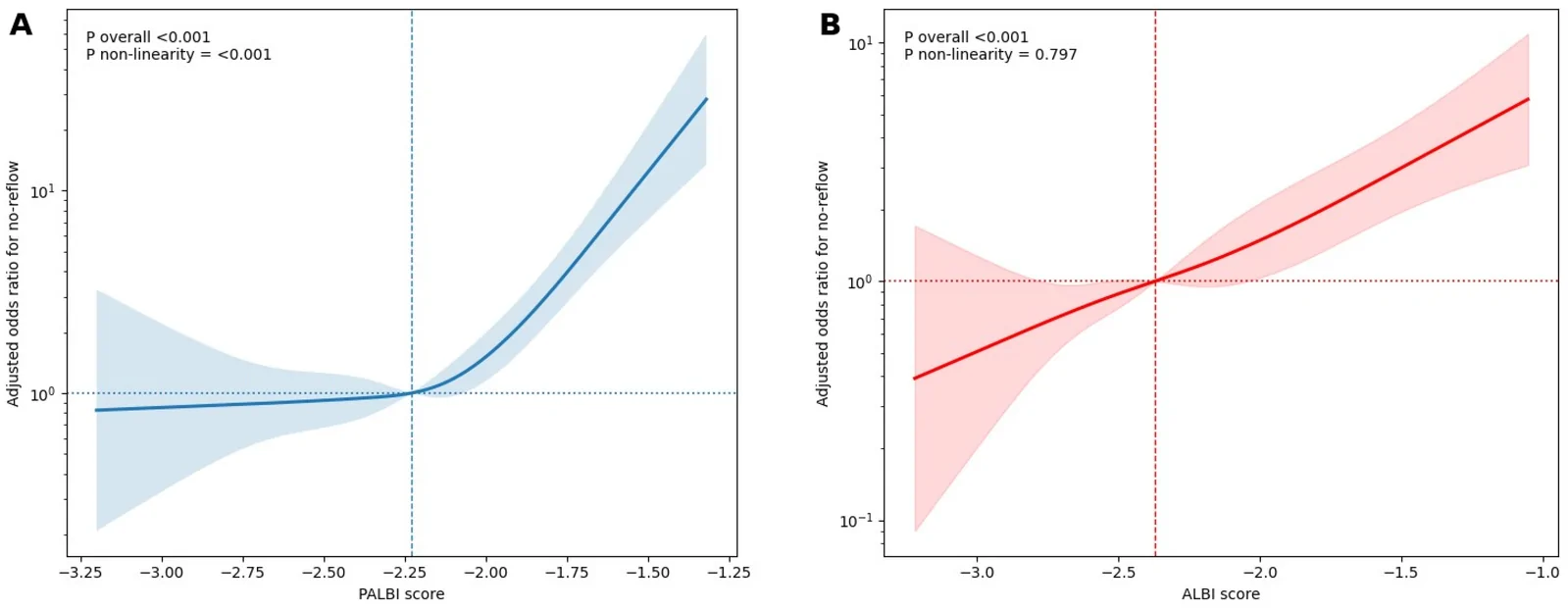
Adjusted restricted cubic spline analyses of the associations between PALBI (**A**) and ALBI (**B**) scores and no-reflow. PALBI showed a significant non-linear association with no-reflow (*p* overall < 0.001; *p* for non-linearity < 0.001), whereas the association for ALBI was significant but predominantly linear (*p* overall < 0.001; *p* for non-linearity = 0.797). Models were adjusted for age, sex, symptom-to-balloon time, LVEF, eGFR, and WBC.

Adjusted RCS analyses demonstrated an overall association between PALBI and no-reflow (*p* overall < 0.001), with evidence of a non-linear relationship (*p* for non-linearity < 0.001) (Figure 5A). The adjusted OR increased progressively at higher PALBI values above the reference level. ALBI was also associated with no-reflow (*p* overall < 0.001); however, there was no evidence of departure from linearity (*p* for non-linearity = 0.797) (Figure 5B).

## 4. Discussion

In this large cohort of patients with STEMI undergoing primary PCI, both PALBI and ALBI were independently associated with angiographic no-reflow after multivariable adjustment. PALBI demonstrated greater discriminative ability than ALBI and provided a more pronounced incremental improvement when added to the clinical reference model. These findings remained consistent across sensitivity analyses and bootstrap internal validation. RCS analysis further demonstrated a non-linear association between PALBI and no-reflow, whereas the association with ALBI was predominantly linear. Overall, both scores provided additional predictive information for no-reflow, with PALBI showing a stronger predictive profile. Of note, symptom-to-balloon time did not reach conventional statistical significance in the univariable analysis but was independently associated with no-reflow after multivariable adjustment. This difference may reflect the distinction between unadjusted and covariate-adjusted estimates, as adjustment for other prognostic factors can alter both the magnitude and precision of an association. The association of male sex with no-reflow in the PALBI-adjusted model also warrants cautious interpretation. Although sex was essentially unrelated to no-reflow in the unadjusted comparison, male sex was independently associated with higher odds of no-reflow in the fully adjusted PALBI model. Exploratory sequential adjustment indicated that this association emerged progressively across the multivariable adjustment process rather than being attributable to the inclusion of a single covariate (Supplementary Table S1). This pattern suggests that the adjusted association reflects the multivariable covariate structure rather than a strong crude association. Given the absence of a significant unadjusted association, the relatively wide confidence interval of the fully adjusted estimate, and differing sex associations reported in previous no-reflow risk models, the magnitude and generalizability of this finding should be interpreted cautiously and warrant further evaluation.

Evidence regarding ALBI in the specific context of coronary no-reflow remains limited. A recent study by Yildirim et al. evaluated 1563 patients with NSTE-ACS undergoing PCI and reported that higher ALBI was independently associated with no-reflow and improved discrimination when incorporated into a multivariable prediction model [15]. Our findings extend these observations to a distinct and clinically important population—patients with STEMI undergoing primary PCI—in whom abrupt coronary occlusion, thrombus burden, prolonged ischemia, distal embolization, and reperfusion injury may result in particularly pronounced microvascular damage [16]. Importantly, our study additionally evaluated PALBI and directly compared the two indices. Although both scores were independently associated with no-reflow, PALBI showed significantly greater discrimination than ALBI and produced a larger improvement in model performance when added to conventional clinical and procedural variables. The significant NRI and IDI observed after adding PALBI to a model already containing ALBI further suggest that the platelet component may capture information not fully represented by albumin and bilirubin alone.

The associations observed in the present study are also biologically plausible when the individual components of these scores are considered. No-reflow is not attributable to a single mechanism but represents the final expression of interacting processes, including distal atherothrombotic embolization, endothelial dysfunction, microvascular obstruction, inflammatory activation, ischemic injury, and reperfusion-mediated oxidative stress [17]. Albumin may be particularly relevant within this framework. Beyond reflecting nutritional and hepatic status, albumin has antioxidant, anti-inflammatory, antithrombotic, and endothelial-stabilizing properties and contributes substantially to plasma oncotic pressure [18]. Lower albumin concentrations may therefore accompany a systemic milieu characterized by greater inflammatory activity, impaired endothelial homeostasis, and increased susceptibility to microvascular injury [19]. Consistent with this concept, albumin concentrations were significantly lower in patients with no-reflow in our cohort.

Bilirubin may provide complementary biological information. Although bilirubin possesses endogenous antioxidant properties at physiological concentrations, increased circulating bilirubin in acute cardiovascular disease may also reflect oxidative stress, altered hepatic perfusion, neurohumoral activation, or systemic illness [20]. A previous study in patients with STEMI demonstrated that higher admission bilirubin concentrations were independently associated with no-reflow after primary PCI [21]. In our population, bilirubin was likewise higher among patients who developed no-reflow. Thus, the combination of lower albumin and higher bilirubin incorporated into ALBI may integrate several systemic processes relevant to microvascular vulnerability rather than merely representing hepatic functional reserve.

The additional platelet component may partly explain the stronger performance of PALBI. Platelets play a central role in the pathobiology of acute STEMI through thrombus formation, platelet–leukocyte interactions, thrombo-inflammatory signaling, and mechanisms contributing to impaired microvascular reperfusion during primary PCI [22,23]. Platelet-related indices and platelet–leukocyte aggregates have previously been associated with impaired myocardial reperfusion in STEMI [24,25], whereas evidence regarding absolute platelet count has been less consistent, with substantial heterogeneity across studies [26]. A recent meta-analysis of laboratory predictors of no-reflow after primary PCI also identified a significant association between platelet count and no-reflow risk [7]. PALBI should therefore not be interpreted simply as a hepatic score with an additional hematological variable. Rather, by mathematically integrating albumin, bilirubin, and platelet count, it may capture overlapping inflammatory, thrombotic, metabolic, and systemic stress signals relevant to the coronary microcirculation. This interpretation remains mechanistic and requires prospective validation, but it offers a plausible explanation for the stronger association and incremental performance observed for PALBI in the present analysis.

**Figure 6:**
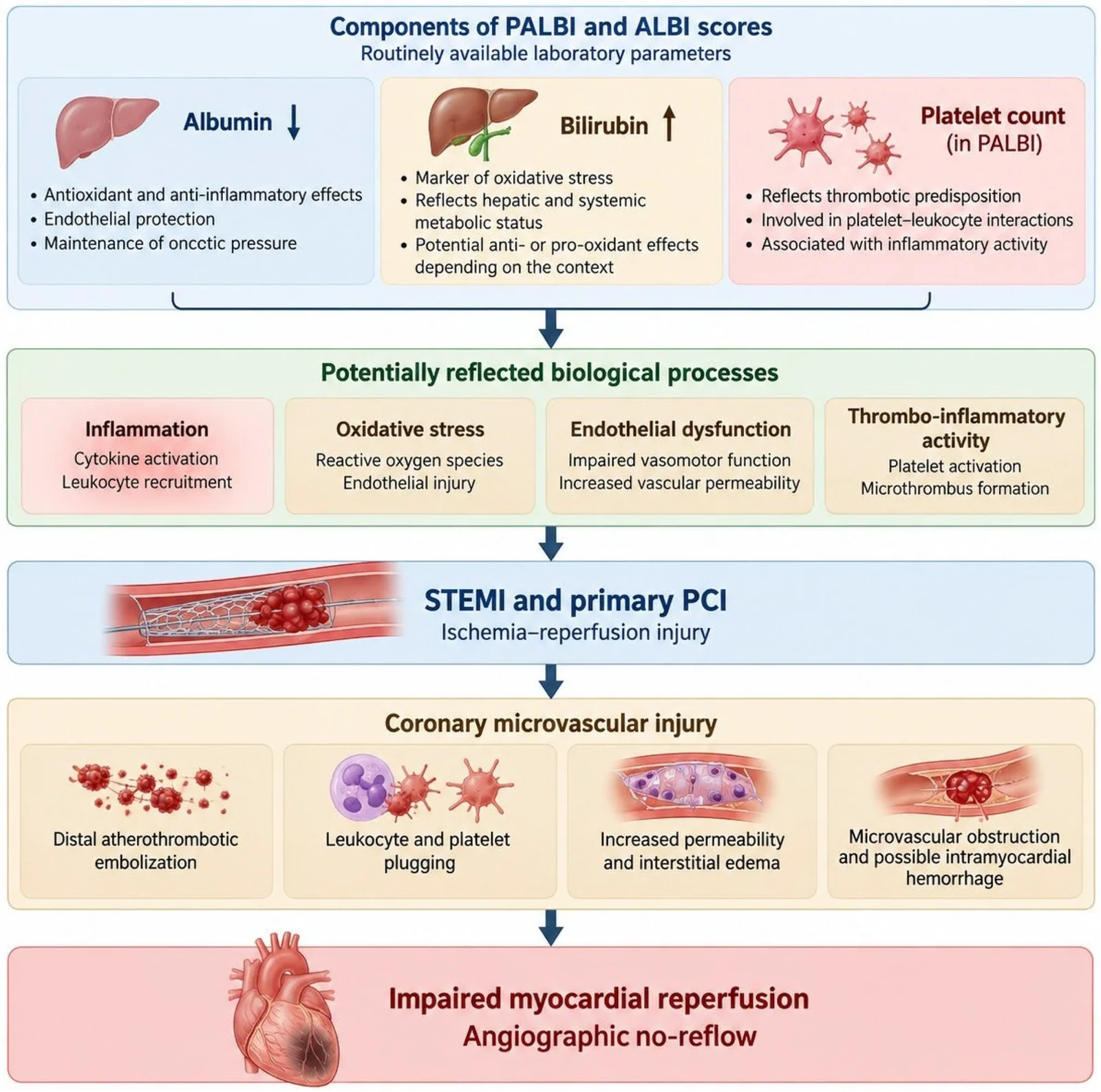
Proposed pathophysiological framework potentially linking the components of the PALBI score with coronary no-reflow after primary PCI. The schematic represents a hypothesis-generating interpretation based on the present findings and the existing literature and does not imply causal relationships demonstrated by this study. PALBI, platelet–albumin–bilirubin; ALBI, albumin–bilirubin; STEMI, ST-segment elevation myocardial infarction; PCI, percutaneous coronary intervention.

At the microvascular level, these processes are likely to converge through an interconnected ischemia–reperfusion cascade rather than acting independently. Prolonged ischemia and subsequent reperfusion can promote endothelial injury and swelling, impaired endothelial-dependent vasomotor function, increased vascular permeability, and interstitial edema, thereby compromising capillary perfusion [27,28]. Reperfusion may further aggravate microvascular injury through a rapid increase in reactive oxygen species, calcium overload, inflammatory activation, and endothelial dysfunction [28]. In parallel, leukocyte and platelet recruitment, platelet–leukocyte interactions, microvascular plugging, and distal atherothrombotic embolization may produce additional intraluminal obstruction and further impair tissue-level reperfusion [27,29]. Increased microvascular permeability and tissue edema may additionally compress the microcirculation, whereas severe structural microvascular injury can culminate in capillary disruption and intramyocardial hemorrhage, representing a particularly advanced form of reperfusion-related microvascular damage [27,30]. Thus, angiographic no-reflow may be a downstream manifestation of interacting endothelial, inflammatory, thrombotic, and structural microvascular abnormalities rather than a consequence of a single pathological pathway [27,28]. Within this framework, PALBI may be particularly informative because its three components could collectively reflect systemic vulnerability across several of these interconnected biological domains; however, this interpretation remains hypothesis-generating, and the present observational data cannot establish a direct mechanistic relationship. A hypothesis-generating framework summarizing the potential biological pathways linking the components of the PALBI and ALBI scores with coronary microvascular injury and angiographic no-reflow is presented in Figure 6.

An additional finding was the different shape of the associations identified by RCS analysis. ALBI showed an approximately linear relationship with no-reflow across its observed range, whereas PALBI demonstrated significant non-linearity, with the odds of no-reflow increasing more steeply at higher PALBI values. This pattern suggests that the information represented by PALBI may not increase uniformly across its distribution and that patients at the higher end of the score range may represent a phenotype with disproportionately greater microvascular vulnerability. Nevertheless, because the present study was not designed to derive or validate a clinical decision threshold, these spline findings should be interpreted as evidence of the shape of the association rather than as support for a specific PALBI cutoff.

Taken together, these observations provide a rationale for evaluating PALBI and ALBI as composite indices rather than viewing their constituent laboratory parameters only in isolation. Importantly, neither score should be interpreted as providing biological information independent of its components; rather, they integrate information captured by routinely available laboratory parameters into predefined composite indices. In an additional component-level analysis, addition of platelet count, albumin, and total bilirubin separately to the clinical reference model increased the AUC from 0.688 to 0.703, 0.710, and 0.761, respectively. Addition of ALBI yielded an AUC of 0.760, whereas PALBI yielded the largest numerical improvement in discrimination, increasing the AUC to 0.785 (Supplementary Table S3). These findings indicate that the predictive signals represented by PALBI and ALBI are derived from information contained in their constituent laboratory parameters, while PALBI provided the largest numerical incremental discrimination among the individual and composite laboratory measures evaluated.

Beyond discrimination alone, the incremental analyses provided complementary evidence regarding the predictive contribution of PALBI and ALBI. Both scores improved reclassification metrics when added to the clinical reference model, although PALBI consistently showed the stronger incremental predictive profile. Notably, the Youden-derived PALBI threshold yielded a negative predictive value of 95.0% in the present cohort. However, predictive values are prevalence-dependent, and this data-derived threshold should not be interpreted as a clinically actionable cutoff without external validation and prospective assessment of clinical utility. Improvements in category-free NRI and IDI were directionally consistent with the ROC findings, and the lower Brier score of the PALBI-augmented model indicated improvement in overall prediction error. Furthermore, bootstrap internal validation showed that the relative advantage of the PALBI-containing model persisted after correction for optimism. Taken together, these complementary analyses suggest that the observed contribution of PALBI was not restricted to a single performance metric. The incremental predictive findings should nevertheless be interpreted in the context of the composition of the clinical reference model. Although this model incorporated multiple clinically relevant demographic, clinical, laboratory, and procedural variables, several established determinants of no-reflow, including baseline TIMI flow, thrombus burden, Killip class/cardiogenic shock, and detailed lesion characteristics [8,31,32], were not systematically available in the retrospective dataset and could not be incorporated. Therefore, the incremental predictive performance observed for PALBI and ALBI should be confirmed against more comprehensive clinical and angiographic reference models in external cohorts.

Importantly, the present findings should not be interpreted as establishing PALBI- or ALBI-guided management. Although both scores are derived from routinely obtained admission laboratory measurements without requiring additional blood sampling or specialized testing, their practical use in the time-sensitive setting of primary PCI would require all component results to be available without delaying reperfusion. In our cohort, the blood samples used for score calculation were collected at emergency department admission before primary PCI; however, the retrospective records did not permit reliable determination of whether all component results were available to the treating interventional cardiologist before initiation of the procedure. Accordingly, the present findings should primarily be viewed as demonstrating an association between the admission laboratory profile represented by PALBI and ALBI and subsequent angiographic no-reflow, rather than establishing these scores as real-time preprocedural decision tools. The present study also did not evaluate whether knowledge of either score would alter preprocedural or periprocedural management or improve clinical outcomes. Thus, PALBI and ALBI should presently be regarded as investigational risk-stratification markers rather than clinically actionable tools. Prospective studies confirming timely preprocedural availability and evaluating predefined score-guided strategies and net clinical benefit are required before either score can be considered for clinical decision-making.

### Study Limitations

Several limitations should be acknowledged. First, the retrospective, single-center design inherently carries the potential for selection bias, residual confounding, and unmeasured confounding and may limit the generalizability of our findings to other STEMI populations and treatment settings. Second, an important limitation concerns the clinical actionability of PALBI and ALBI in the time-sensitive setting of primary PCI. Although the blood samples used for score calculation were collected at emergency department admission before primary PCI, the retrospective records did not permit reliable determination of whether all component laboratory results were available to the treating interventional cardiologist before initiation of the procedure. Moreover, the present study did not evaluate whether knowledge of PALBI or ALBI before PCI would alter preprocedural or periprocedural management, reduce the occurrence of no-reflow, or improve clinical outcomes. Therefore, the present findings establish predictive associations but do not establish a clinically actionable role for either score. Prospective studies demonstrating timely preprocedural availability and evaluating predefined score-guided management strategies and net clinical benefit would be required before either score could be considered for clinical decision-making. Third, although patients with known chronic liver disease were excluded, the retrospective design did not allow for systematic assessment of non-alcoholic fatty liver disease (NAFLD), currently termed metabolic dysfunction-associated steatotic liver disease (MASLD), because dedicated hepatic imaging and other diagnostic information required to ascertain steatotic liver disease were not consistently available. Therefore, unrecognized NAFLD/MASLD and its potential influence on the observed associations cannot be excluded. Fourth, no-reflow was retrospectively ascertained from procedural records based on TIMI flow and MBG assessments performed by the treating interventional cardiologists, without independent core-laboratory adjudication. Although this approach reflects routine clinical practice, interobserver variability cannot be excluded. In addition, although angiographic no-reflow was defined using the composite criterion of final TIMI flow < 3 and/or MBG 0–1, the individual final TIMI flow and MBG components were not retained as separate analyzable variables in the retrospective dataset. Therefore, component-specific analyses distinguishing persistent impairment of epicardial flow from impaired myocardial blush despite preserved epicardial flow could not be performed. Future studies with separately recorded TIMI flow and MBG data are needed to determine whether the observed associations with PALBI and ALBI are consistent across these distinct angiographic manifestations of impaired reperfusion. Fifth, despite adjustment for multiple clinically relevant variables and the consistency of the findings across several sensitivity models, residual confounding from unmeasured angiographic, procedural, pharmacological, or biological factors remains possible. In particular, some established angiographic and clinical determinants of no-reflow were not systematically available in the retrospective dataset and therefore could not be incorporated into the reference model. This limitation should be considered when interpreting the magnitude of the incremental predictive improvement associated with PALBI and ALBI. Sixth, although bootstrap internal validation was performed to quantify optimism and assess model stability, external validation in an independent cohort was not available. Accordingly, the predictive performance and clinical applicability of PALBI and ALBI should be confirmed in prospective, multicenter studies before their incorporation into routine risk-stratification strategies. Finally, the observational nature of the study precludes causal inference regarding the relationship between these indices and the development of no-reflow.

## 5. Conclusions

In patients with STEMI undergoing primary PCI, higher PALBI and ALBI scores were independently associated with angiographic no-reflow. PALBI demonstrated a stronger predictive profile than ALBI and provided incremental predictive information beyond conventional clinical and procedural variables. These findings identify PALBI as a candidate marker for further investigation of no-reflow risk in STEMI. External validation and prospective studies evaluating clinical utility and net benefit are required before PALBI can be considered for routine risk stratification or clinical decision-making.

## Figures and Tables

**Table 1:** Clinical, laboratory, angiographic, procedural, and in-hospital characteristics according to no-reflow status.

Variable	No-Reflow (−) (*n* = 1393)	No-Reflow (+) (*n* = 146)	*p* Value
Age, years	58.00 [50.00–66.00]	60.00 [52.00–67.75]	0.064
Male sex, *n* (%)	1171 (84.1)	124 (84.9)	0.877
Cardiac arrest on admission, *n* (%)	56 (4.1)	8 (5.6)	0.526
Smoking, *n* (%)	609 (43.7)	67 (45.9)	0.678
Comorbidities, *n* (%)			
Coronary artery disease history	211 (15.1)	26 (17.8)	0.467
PCI history	184 (13.2)	19 (13.0)	1.000
Heart failure	65 (4.7)	11 (7.5)	0.187
Diabetes mellitus	338 (24.3)	37 (25.3)	0.851
Hypertension	438 (31.4)	49 (33.6)	0.667
Hyperlipidemia	334 (24.0)	29 (19.9)	0.312
Chronic kidney disease	23 (1.7)	8 (5.5)	0.006
Cerebrovascular disease	20 (1.4)	5 (3.4)	0.081
COPD	13 (0.9)	2 (1.4)	0.646
Anemia	367 (26.3)	40 (27.4)	0.861
Admission laboratory findings			
LVEF, %	50.00 [40.00–55.00]	45.00 [39.00–55.00]	0.046
White blood cells, 10^3^/mm^3^	11.40 [9.30–13.90]	12.20 [9.76–16.62]	0.001
Hemoglobin, g/dL	13.80 [12.60–14.90]	13.70 [12.80–14.80]	0.837
Platelet, 10^3^/mm^3^	221.00 [188.00–258.00]	204.00 [170.50–255.75]	0.016
Neutrophil, 10^3^/mm^3^	8.82 [6.69–11.20]	9.05 [7.06–12.57]	0.114
Lymphocyte, 10^3^/mm^3^	1.50 [1.10–2.20]	1.50 [1.10–2.30]	0.973
eGFR, mL/dk/1.73 m^2^	92.30 [76.70–102.00]	86.80 [60.15–98.95]	<0.001
Serum creatinine, mg/dL	0.88 [0.78–1.00]	0.95 [0.80–1.20]	<0.001
Blood glucose, mg/dL	132.00 [112.00–171.00]	142.00 [114.25–189.75]	0.059
Aspartate aminotransferase, U/L	60.00 [30.00–130.00]	87.00 [30.25–166.50]	0.138
Alanine aminotransferase, U/L	31.00 [20.00–47.00]	34.00 [23.00–56.75]	0.105
Serum sodium, mmol/L	136.00 [134.00–139.00]	137.00 [134.00–139.00]	0.729
Serum albumin, g/dL	3.70 [3.30–3.90]	3.40 [3.10–3.88]	<0.001
Total bilirubin, mg/dL	0.79 [0.60–1.00]	1.54 [0.88–2.21]	<0.001
Triglyceride, mg/dL	136.00 [97.00–187.00]	134.00 [97.00–186.00]	0.977
HDL cholesterol, mg/dL	37.00 [31.00–43.00]	38.00 [30.25–43.00]	0.649
LDL cholesterol, mg/dL	110.00 [86.00–137.00]	104.50 [85.50–136.25]	0.446
C-reactive protein, mg/L	7.00 [4.00–9.00]	7.00 [5.00–9.00]	0.202
PALBI score	−2.26 [−2.47–−2.07]	−1.87 [−2.31–−1.64]	<0.001
ALBI score	−2.39 [−2.65–−2.08]	−2.08 [−2.44–−1.60]	<0.001
In-hospital biomarker			
Peak troponin, ng/mL	45.44 [16.67–50.00]	49.87 [16.67–50.00]	0.785
Pre-admission medications, *n* (%)			
Antiplatelet	356 (25.6)	30 (20.5)	0.219
Beta-blocker	328 (24.1)	33 (23.6)	0.968
ACEi or ARB	450 (33.0)	45 (31.7)	0.817
Statin	527 (39.8)	52 (38.5)	0.848
Antidiabetic	345 (24.8)	37 (25.3)	0.878
Periprocedural details			
Symptom-to-balloon time, min	180.00 [120.00–300.00]	210.00 [97.50–345.00]	0.308
Culprit vessel, *n* (%)			0.056
LAD	615 (44.1)	51 (34.9)	
LCx	211 (15.1)	36 (24.7)	
RCA	535 (38.4)	56 (38.4)	
OM	19 (1.4)	2 (1.4)	
Diagonal	7 (0.5)	1 (0.7)	
Intermediate artery	6 (0.4)	0 (0.0)	
Patients receiving tirofiban, *n* (%)	727 (52.2)	77 (52.7)	0.968
Multivessel disease, *n* (%)	271 (19.5)	29 (19.9)	0.993
Stent diameter, mm	3.00 [2.75–3.50]	3.00 [2.75–3.50]	0.243
Stent length, mm	20.00 [16.00–24.00]	20.00 [18.00–28.00]	0.004
In-hospital mortality, *n* (%)	100 (7.2)	20 (13.7)	0.005
Length of in-hospital stay, days, median (IQR)	3 (3–4)	3 (3–4)	0.122

Notes: Continuous variables: median [IQR], Mann–Whitney U test. Categorical variables: *n* (%), Pearson chi-square or Fisher’s exact test, as appropriate. With the exception of peak troponin, laboratory variables were measured from blood samples obtained at emergency department admission before primary PCI. Peak troponin represents the highest troponin concentration recorded during serial measurements over the index hospitalization and should not be interpreted as a baseline measurement. Abbreviations: ACEi, angiotensin-converting enzyme inhibitor; ALBI, albumin–bilirubin; ARB, angiotensin receptor blocker; COPD, chronic obstructive pulmonary disease; eGFR, estimated glomerular filtration rate; HDL, high-density lipoprotein; LAD, left anterior descending artery; LDL, low-density lipoprotein; LCx, left circumflex artery; LVEF, left ventricular ejection fraction; OM, obtuse marginal artery; PALBI, platelet–albumin–bilirubin; PCI, percutaneous coronary intervention; RCA, right coronary artery.

**Table 2:** Univariable logistic regression analysis for no-reflow.

Variable	Odds Ratio	95% CI	*p* Value
PALBI score, per 0.1-point increase	1.296	1.230–1.358	<0.001
ALBI score, per 0.1-point increase	1.143	1.104–1.182	<0.001
Symptom-to-balloon time, per 30 min	1.030	0.995–1.067	0.095
Age, per 10 years	1.153	1.000–1.330	0.050
LVEF, per 5%	0.904	0.830–0.986	0.022
WBC, per 1 × 10^3^/mm^3^	1.072	1.037–1.108	<0.001
eGFR, per 10 mL/min/1.73 m^2^	0.838	0.779–0.901	<0.001
Serum creatinine, per 1 mg/dL	3.032	1.958–4.695	<0.001
Blood glucose, per 10 mg/dL	1.022	1.004–1.041	0.016
Serum albumin, per 1 g/dL	0.530	0.391–0.719	<0.001
Total bilirubin, per 0.1 mg/dL	1.431	1.355–1.510	<0.001
Stent length, per 5 mm	1.215	1.050–1.406	0.009
Chronic kidney disease	3.453	1.516–7.866	0.003
Culprit vessel (overall)			0.061
LCx vs. LAD	2.057	1.306–3.241	0.002

Only variables with *p* < 0.10 in univariable logistic regression are presented. Abbreviations: ALBI, albumin–bilirubin; CI, confidence interval; eGFR, estimated glomerular filtration rate; LAD, left anterior descending artery; LCx, left circumflex artery; LVEF, left ventricular ejection fraction; PALBI, platelet–albumin–bilirubin; WBC, white blood cell count.

**Table 3:** Multivariable logistic regression analyses for no-reflow.

Variable	Model 1—PALBI			Model 2—ALBI		
	aOR	95% CI	*p* Value	aOR	95% CI	*p* Value
PALBI score, per 0.1 increase	1.315	1.228–1.408	<0.001	-	-	-
ALBI score, per 0.1 increase	-	-	-	1.165	1.115–1.218	<0.001
Age, per 10 years	1.108	0.878–1.398	0.389	1.078	0.860–1.352	0.513
Male sex	2.325	1.117–4.842	0.024	1.892	0.927–3.862	0.080
Symptom-to-balloon time, per 30 min	1.063	1.017–1.111	0.007	1.063	1.017–1.110	0.007
LVEF, per 5%	0.958	0.855–1.075	0.467	0.967	0.865–1.082	0.557
eGFR, per 10 mL/min/1.73 m^2^	0.895	0.789–1.014	0.082	0.882	0.782–0.995	0.042
WBC, per 1 × 10^3^/mm^3^	1.039	0.990–1.090	0.121	1.063	1.015–1.114	0.010
Stent length, per 5 mm	1.189	0.996–1.419	0.055	1.149	0.968–1.364	0.112
Blood glucose, per 10 mg/dL	0.994	0.966–1.023	0.668	0.991	0.964–1.019	0.531
LCx vs. LAD	1.936	1.060–3.535	0.031	2.360	1.308–4.258	0.004

Note: Variables with a *p* value <0.10 in univariable analysis were considered for inclusion in the multivariable models. Serum creatinine and chronic kidney disease were excluded because of multicollinearity with eGFR. Serum albumin and total bilirubin were not entered into the multivariable models because they are components of the PALBI and/or ALBI scores, thereby avoiding mathematical coupling and collinearity with the investigated scores. PALBI and ALBI were evaluated in separate multivariable models. Abbreviations: ALBI, albumin–bilirubin; aOR, adjusted odds ratio; CI, confidence interval; eGFR, estimated glomerular filtration rate; LAD, left anterior descending artery; LCx, left circumflex artery; LVEF, left ventricular ejection fraction; PALBI, platelet–albumin–bilirubin; WBC, white blood cell count.

**Table 4:** Sensitivity analyses of the association of PALBI and ALBI scores with no-reflow across different adjustment models.

Model	PALBI			ALBI			Max VIF *
	aOR/0.1	95% CI	*p* Value	aOR/0.1	95% CI	*p* Value	
Model A	1.296	1.230–1.366	<0.001	1.140	1.101–1.179	<0.001	1.108
Model B	1.307	1.238–1.379	<0.001	1.140	1.101–1.180	<0.001	1.174
Model C	1.309	1.230–1.393	<0.001	1.156	1.110–1.204	<0.001	1.199
Model D	1.292	1.221–1.367	<0.001	1.140	1.099–1.183	<0.001	1.123
Model E	1.335	1.248–1.428	<0.001	1.171	1.121–1.223	<0.001	1.289

Adjustment models: Model A: Age + sex. Model B: Age + sex + diabetes + smoking + cardiac arrest on admission. Model C: Age + sex + blood glucose + WBC + triglyceride. Model D: Age + sex + chronic kidney disease + symptom-to-balloon time + LVEF. Model E: Age + sex + symptom-to-balloon time + LVEF + stent length + culprit vessel (LAD reference). * Maximum VIF is shown across variables in each PALBI/ALBI model pair; all values were <1.30, indicating no relevant multicollinearity. Abbreviations: ALBI, albumin–bilirubin; aOR, adjusted odds ratio; CI, confidence interval; LAD, left anterior descending artery; LVEF, left ventricular ejection fraction; PALBI, platelet–albumin–bilirubin; VIF, variance inflation factor; WBC, white blood cell count.

## Data Availability

Data are available from the corresponding author upon reasonable request. The data are not publicly available due to privacy or ethical restrictions.

## Supplementary Materials

The following supporting information can be downloaded at: https://www.mdpi.com/article/10.3390/jcm15207871/s1, Table S1: Exploratory sequential adjustment analysis of the association between male sex and angiographic no-reflow; Table S2: Sensitivity analysis using a presentation-based reference model excluding angiographic and procedural variables; Table S3: Incremental discriminative performance of individual laboratory components and the composite PALBI and ALBI scores when added separately to the base clinical model.
