# Supplementary material for: Association of Platelet–Albumin–Bilirubin and Albumin–Bilirubin Scores with Angiographic No-Reflow in Patients with ST-Segment Elevation Myocardial Infarction Undergoing Primary PCI
Source: J Clin Med. 2026 Oct 12;15(20):7871. doi: 10.3390/jcm15207871 (PMC13705421; doi:10.3390/jcm15207871)
Supplement: Supplementary file 1 [file jcm-15-07871-s001.zip › jcm-4529348-supplementary.pdf]

Supplementary Table S1. Exploratory sequential adjustment analysis of the association between male sex and angiographic no-reflow.

Sequential model	Male sex OR	95% CI	p-value
**Sex only**	1.316	0.703–2.464	0.391
**+ PALBI**	1.566	0.807–3.039	0.185
**+ Age**	1.845	0.935–3.641	0.077
**+ Symptom-to-balloon time**	1.949	0.980–3.874	0.057
**+ Left ventricular ejection fraction**	1.915	0.963–3.809	0.064
**+ Estimated glomerular filtration rate**	2.202	1.081–4.484	0.030
**+ White blood cell count**	2.192	1.073–4.476	0.031
**+ Blood glucose**	2.159	1.053–4.430	0.036
**+ Stent length**	2.194	1.066–4.517	0.033
**+ Culprit vessel (Final model)**	2.325	1.117–4.842	0.024

Note: Sequential models were constructed as an exploratory analysis to examine the change in the estimated association between male sex and angiographic no-reflow across progressive covariate adjustment. The final model corresponds to the fully adjusted PALBI model reported in **Table 3**. The sequence of covariate entry was used for descriptive exploration of coefficient changes and should not be interpreted as attributing the observed change in the sex estimate to any individual covariate.

Supplementary Table S2. Sensitivity analysis using a presentation-based reference model excluding angiographic and procedural variables.

Model	Adjusted OR for score (95% CI)*	p-value for score	AUC (95% CI)	ΔAUC vs. reference model	DeLong p value	Brier score
**Presentation-based reference model**	—	—	0.623 (0.572–0.675)	—	—	0.083
**Presentation-based reference model + ALBI**	1.129 (1.090–1.169)	<0.001	0.711 (0.665–0.756)	+0.087	<0.001	0.080
**Presentation-based reference model + PALBI**	1.273 (1.206–1.343)	<0.001	0.745 (0.697–0.792)	+0.121	<0.001	0.076

Note: The presentation-based reference model included age, sex, estimated glomerular filtration rate, white blood cell count, and admission blood glucose. Variables determined during angiography or PCI, including culprit vessel and stent length, as well as symptom-to-balloon time, which is fully determined only at reperfusion, were intentionally excluded. Analyses were performed in 1,537 patients with complete data for all variables included in the presentation-based model. *Adjusted ORs for ALBI and PALBI are expressed per 0.1-point increase in the respective score and were adjusted for all variables included in the presentation-based reference model. The AUC of the PALBI-augmented model was also significantly greater than that of the ALBI-augmented model (ΔAUC=0.034; DeLong p=0.029). ΔAUC represents the change in AUC after addition of ALBI or PALBI to the presentation-based reference model; p-values for ΔAUC were calculated using DeLong's test. Brier scores are reported as an overall measure of prediction error.

Supplementary Table S3. Incremental discriminative performance of individual laboratory components and the composite PALBI and ALBI scores when added separately to the base clinical model.

Model	Parameters	AUC (95% CI)	ΔAUC (vs Base)	DeLong p (vs Base)
X	**Base model only**	0.688 (0.627–0.741)	—	—
Y	**Base + Platelet**	0.703 (0.649–0.758)	+0.015	0.102
Z	**Base + Albumin**	0.710 (0.658–0.764)	+0.022	0.092
T	**Base + Total bilirubin**	0.761 (0.711–0.809)	+0.073	0.002
W	**Base + PALBI**	0.785 (0.733–0.835)	+0.098	0.001
V	**Base + ALBI**	0.760 (0.709-0.808)	+0.073	0.003

Note: The base clinical model included age, sex, left ventricular ejection fraction, estimated glomerular filtration rate, white blood cell count, glucose, symptom-to-balloon time, stent length, and culprit vessel. Each individual laboratory parameter or composite score was added separately to the same base clinical model. ΔAUC represents the change in AUC relative to the base model; p values were derived from DeLong comparisons with the base model.
